# Medical security and catastrophic health expenditures among households containing persons with disabilities in Korea: a longitudinal population-based study

**DOI:** 10.1186/s12939-016-0406-9

**Published:** 2016-07-26

**Authors:** Jae Woo Choi, Jae-Yong Shin, Kyung-Hee Cho, Jin-Young Nam, Ju-Young Kim, Sang Gyu Lee

**Affiliations:** 1 Department of Public Health, Graduate School, Yonsei University, Seoul, Korea; 2 Institute of Health Services Research, Yonsei University College of Medicine, Seoul, Korea; 3 Department of Preventive Medicine, Yonsei University College of Medicine, Seoul, Korea; 4 Department of Hospital Management, Graduate School of Public Health, Yonsei University, 50 Yonsei-ro, Seodaemun-gu, Seoul 120-752 Republic of Korea

**Keywords:** Persons with disability, Medical-Aid, Catastrophic health expenditure, Blind spot

## Abstract

**Background:**

Although persons with disabilities need access to comprehensive and consistent healthcare services, a significant number of the poor with disabilities do not receive Medical-Aid due to the conditions of eligibility. We aimed to compare the financial burden of healthcare services between two groups of poor persons with disabilities: those not enrolled in Medical-Aid and Medical-Aid enrollees.

**Methods:**

This study used the 1st–8th data (2008–2014 year) of Panel Survey of Employment for the Disabled (PSED) conducted by the Korea Employment Agency for the Disabled. We classified adults who did not exceed 100 % of the poverty level into two groups (*N* = 3,010). The first group consisted of enrollees in Medical-Aid (*n* = 1,259) and the second group comprised those not enrolled in Medical-Aid (*n* = 1,325). We applied generalized estimating equations (GEEs) to assess the independent effect of enrollment in Medical-Aid on catastrophic health expenditures (CHE).

**Results:**

We found that about 4.2 % of the poor not enrolled in the Medical-Aid experienced CHE and the poor not enrolled in Medical-Aid were 2.1 times more likely to experience CHE than Medical-Aid enrollees after applying multivariate models adjusted for several covariates.

**Conclusions:**

Given the additional expenses for treatment and rehabilitation caused by disability-related health problems, persons with disabilities are more likely to face barriers to needed medical services. Thus, policy makers need to expand the number of people receiving Medical-Aid by loosening the strict criteria for those with disabilities.

## Background

The number of registered disabled has increased continuously in Korea (1,610,994 in 2004 to 2,494,460 in 2014), and persons with disabilities accounted for 4.9 % of the total population in 2014. Commensurate with growing welfare demand, the government has provided tailored services for rehabilitation and self-reliance support so that persons with disabilities can live in comfort and participate in social activities [1].

However, persons with disabilities have difficulties in getting jobs, due to low education and lack of ability as well as discrimination against the disabled in our society [2]. Even when they do get a job, they often work under poor conditions and in temporary capacities. This means that they may fall into poverty because of relatively low income levels and the additional costs associated with disabilities [3–7]. The monthly average income level of household with persons with disabilities was reported as approximately US $1,667, which was 53.4 % of the national monthly average income level per household in Korea [8].

Persons with disabilities need access to comprehensive and consistent healthcare services because they often experience disability-related health problems in addition to common health conditions not associated with their disability [9, 10]. That is, their medical expenditures are consistently higher than those for persons without a disability [11, 12]. The proportion of persons with a disability who had incurred additional disability-based expenses in 2013 was 72 %, and their monthly average additional expenditure was US $139. Thus, medical expenses accounted for the highest proportion of their income [8].

To protect persons with disabilities, the government operates the Medical-Aid program, which is a public assistance program targeted at poor individuals who are recipients of the National Basic Livelihood Security System (NBLSS) in Korea as part of the social welfare program. The NBLSS is the pivotal channel for providing income security for people living below the poverty line. The NBLSS brought a paradigm shift in the public assistance system of Korea by emphasizing social responsibility for poverty and strengthening a rights-based approach to public assistance. In the 40 years prior to the introduction of NBLSS, the Korean government provided limited protection to disadvantaged households, and mainly aided those unable to work due to age or disabilities under the previous Livelihood Protection Act of 1961. However, the Korean financial crisis in 1990s caused a rapid increase in the number of poor and unemployed persons, which required urgent expansion of the social safety net. To address these problems, the government enacted the National Basic Livelihood Security Act in 1999, and fully implemented the system in October 2000. NBLSS had 1,329 thousands beneficiaries, representing 2.6 % of the country’s population in 2014. The beneficiaries are supported by seven kinds of benefits, including Medical-Aid [13]. Medical-aid had 1,507,044 beneficiaries, representing 3.0 % of the country’s population, in 2012 and Medical-Aid provides assistance to the poor with almost-free medical services [14]. The Korean Medical-Aid program is comparable to the US “Medicaid” program, which was established in 1965, and provided healthcare services to approximately 58 million people in 2011, including low-income families, seniors, disabled, and pregnant women [15].

However, a significant number of low-income persons with disabilities that enrolled in government publicly do not receive Medical-Aid due to the conditions of eligibility in individual level. Even if their income level is below 100 % of the poverty line, which is the minimum cost of living, they are excluded when the value of their property is above a certain threshold, or the income property of their support obligor is over a certain level [14]. Minimum cost of living is the minimum expense to sustain one’s life. The 2014 minimum monthly cost of living presented by the government was 503 USD per single-person household, 856 USD per two-person household, 1,108 USD per three-person household, and 1,359 USD per four-person household [16]. A previous study showed that poor households with persons with disabilities supported by Medical-Aid accounted for 56.7 % of the total target population [17]. Thus, households that are excluded from public assistance may experience a higher burden of medical costs compared with Medical-Aid enrollees. However, no empirical research examining catastrophic health expenditures (CHE) among persons with disabilities has been conducted.

Thus, in this study, we aim to compare the financial burden of healthcare services between two groups of poor persons with disabilities: those not enrolled in Medical-Aid and Medical-Aid enrollees. We also compared these groups in analyses stratified for chronic disease and disability severity status.

## Methods

### Data

This study used the 1st–8th data (2008–2014 year) of Panel Survey of Employment for the Disabled (PSED) conducted by the Korea Employment Agency for the Disabled. The PSED provides panel data from repeatedly measured households containing persons with disabilities that include the demographic and socio-economic characteristics of individuals, factors related to disabilities, and household variables (e.g., income and expenditure, including medical costs). PSED directly carried out 1:1 interview surveys with persons with disabilities and used a computer-assisted personal interviewing (CAPI) method to collect accurate information. Interviewers entered the responses of interviewees in the computer-installed CAPI and investigators were able to check logically incorrect responses using the method. The PSED only allowed the head of the household or nearest guardian to reply if a direct response was impossible due to an intellectual disability or mental disorder. The average substitute response rate was 7.0 % and the follow-up loss rate was 81.0 % over 7 years.

### Study sample

**Figure 1:**
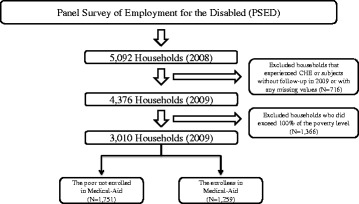
Flow chart for selection of study subjects

This study included data from the first wave of surveyed PSED households (2008). In the first wave, 5,092 subjects completed the survey questionnaire. The baseline study subjects were households living with persons with disabilities who did not experience CHE. After excluding subjects without follow-up in 2009 or with any missing values, a total of 3,010 households living with persons with disabilities remained in the study (Fig. 1).

We classified adults who did not exceed 100 % of the poverty level, which is the minimum cost of living and is measured by equalized income, into two groups (*N* = 3,010). The first group consisted of enrollees in Medical-Aid (*n* = 1,259) and the second group comprised those not enrolled in Medical-Aid (*n* = 1,325).

### Dependent variable

The dependent variable was CHE, as defined by the World Health Organization (WHO). According to the WHO, CHE occurs when out-of-pocket (OOP) spending exceeds 40 % of a household’s capacity to pay; this standard should be altered as appropriate for each country. We defined a household’s capacity to pay as the amount of money spent per month, excluding food expenses [18, 19]. An OOP expenditure is a payment made by a household when receiving a health service. Medical and drug costs resulting from emergency and outpatient care, as well as from hospitalizations, were included as OOP payments, whereas transportation and nursing costs were excluded. Thus, CHE was defined as health expenditures that were 40 % greater than the capacity of the household to pay.

### Independent variable

We compared the burden of medical costs of the Medical-Aid-enrolled and non-enrolled groups. The Medical-Aid enrollees receive assistance in the form of almost-free medical services, whereas the poor not enrolled in Medical-Aid have to pay regular prices for their treatments.

### Covariates

We used several covariates to analyze demographic and socioeconomic characteristics and health status. Demographic characteristics included age, gender, marital status, and socioeconomic factors, such as education level. As a proxy for health status, we used severity of disability, self-rated health, and chronic diseases to control for the person’s health-related behavior and overall health condition, both of which can affect medical services utilization. We utilized severity of disability based on objective evidence. People with disabilities receive the judgment for degree of disability from government. Degree of disability is categorized by six levels and first to third disability ratings generally indicate severe disability and the others are mild disability in Korea. We also examined household characteristics, such as the number of disabled people, number of senior citizens, and household income. Income level was measured by summing the income of all the members of the household, including income from pensions, financial support from the government, assets, and other sources. Household income was adjusted by taking the square root of the number of household members to facilitate comparisons between households of differing size and composition, reflecting the requirement of a larger household to have a higher level of income than a smaller household to achieve the same standard of living [20, 21].

## Statistical analysis

General characteristics were analyzed using descriptive statistics and comparisons between the groups were made using t-tests or *χ*^2^ tests. We developed a series of multivariate models to assess the independent effect of enrollment in the Medical-Aid program on CHE. Our basic analysis unit is individual level of person with disabilities and we added household level characteristics because CHE is associated with household-related factors. We adjusted the models for individual- and household-level characteristics and then applied generalized estimating equations (GEEs) to the data using SAS software (ver. 9.3; SAS Institute, Cary, NC, USA). We also used logit-link GEEs to examine the association between the variable of interest and the dependent variable. For all statistical tests, the level of significance was set at 0.05.

## Results

The demographic, socioeconomic, and health related characteristics of the two groups are shown in Table 1. At an individual level, the poor not enrolled in Medical-Aid included a higher percentage of persons aged 60 years and over compared with the Medical-Aid enrollees (22.6 % vs. 16.1 %, respectively, *p* < 0.001). The poor not enrolled in Medical-Aid tended to have a higher level of education (7.4 % vs. 4.5 %, respectively, *p* < 0.001), were more likely to be married (71.2 % vs. 40.7 %, respectively, *p* < 0.001), and had less-severe disabilities (64.0 % vs. 41.0 %, respectively, *p* < 0.001). In addition, they tended to have better self-rated health (39.5 % vs. 24.8 %, respectively, *p* < 0.001), and fewer chronic diseases (52.3 % vs. 41.9 %, respectively, *p* < 0.001). At the household level, the poor not enrolled in Medical-Aid tended to have higher income levels (36.4 % vs. 9.1 %, respectively, *p* < 0.001), were less likely to be disabled (11.9 % vs. 26.1 %, respectively, *p* < 0.001), and were more likely to be senior citizens over 65 years old (19.3 % vs. 13.3 %, respectively, *p* < 0.001). No statistically significant difference in gender ratio was observed between the two groups.

**Table 1:** General characteristics

			Unit: N (%)	
Variable	Total	The poor not enrolled in medical-aid	Enrollees in medical-aid	*p*-value
	*(N = 3,010)*	*(n = 1,751)*	*(n = 1,259)*	
Individual level				
Sex				0.5046
Male	1,909	1,123 (64.1)	786 (62.4)	
Female	1,101	628 (35.9)	473 (37.6)	
Age				<0.001
≤ 39	432	256 (14.6)	176 (14.0)	
40–49	826	418 (23.9)	408 (32.4)	
50–59	1,154	682 (39.0)	472 (37.5)	
≥ 60	598	395 (22.6)	203 (16.1)	
Education level				<0.001
Below elementary school	1,352	745 (42.5)	607 (48.2)	
Middle/high school	1,472	877 (50.1)	595 (47.3)	
Above university	186	129 (7.4)	57 (4.5)	
Marital status				<0.001
Married	1759	1,246 (71.2)	513 (40.7)	
Single	522	226 (12.9)	296 (23.5)	
Divorced or separated	729	279 (15.9)	450 (35.7)	
Severity of disability				<0.001
Low	1,637	1,121 (64.0)	516 (41.0)	
High	1,373	630 (36.0)	743 (59.0)	
Self-rated health				<0.001
Good	1,003	691 (39.5)	312 (24.8)	
Bad	2,007	1,060 (60.5)	947 (75.2)	
Chronic disease				<0.001
Yes	1,566	835 (47.7)	731 (58.1)	
No	1,444	916 (52.3)	528 (41.9)	
Household level				
Equalized income				<0.001
Q1 (lowest income)	746	408 (23.3)	338 (26.8)	
Q2	757	279 (15.9)	478 (38.0)	
Q3	755	427 (24.4)	328 (26.1)	
Q4 (highest income)	752	637 (36.4)	115 (9.1)	
Disabled				<0.001
1	2,474	1,543 (88.1)	931 (73.9)	
≥ 2	536	208 (11.9)	328 (26.1)	
Employed				<0.001
0	1,687	651 (37.2)	1,036 (82.3)	
1	1,323	1,100 (62.8)	223 (17.7)	
Senior citizen				<0.001
0	2,504	1,413 (80.7)	1,091 (86.7)	
≥ 1	506	338 (19.3)	168 (13.3)	

Data on the average capacity to pay, OOP spending, and proportion of CHE of each group (not adjusted for other covariates) are shown in Table 2. Overall, the poor not enrolled in the Medical-Aid program had a higher capacity to pay than the Medical-Aid enrollees ($6,417 vs. $3,398, respectively, *p* < 0.001). However, the OOP spending ($706 vs. $242, respectively, *p* < 0.001) and proportion of CHE (4.2 % vs. 2.6 %, respectively, *p* < 0.05) were greater in the non-Medical-Aid-enrolled versus Medical-Aid-enrolled group.

**Table 2:** Average capacity to pay, OOP spending, and burden of medical costs by group

					Unit: USD^b^
Variable	The poor not enrolled in medical-aid (*n* = 1,751)		Enrollees in medical-aid (*n* = 1,259)		*p*-value^a^
	Mean	Std Dev	Mean	Std Dev	
Capacity to pay	6,417	4,375	3,398	1,962	<0.001
OOP spending	706	1,371	242	456	<0.001
Catastrophic health expenditure (%)	4.2		2.6		0.023

^a^T-test or Chi-square test

^b^1 USD = 1,200 won (2016. 02)

OOP out-of-pocket

After applying multivariate models adjusted for several covariates, the results for factors associated with catastrophic health expenditure are shown in Table 3. The results indicate that persons with negative self-rated health were more likely to experience CHE than those who had positive self-rated health (odds ratio [OR] = 1.85, 95 % CI: 1.07-3.22). Persons with chronic diseases were more likely to experience CHE than those who had no chronic disease (OR = 1.95, 95 % CI: 1.44-2.75). The poor not enrolled in Medical-Aid were more likely to experience CHE than Medical-Aid enrollees (OR = 2.09, 95 % CI: 1.20-3.65). Households with more than two disabled persons were 1.7 times more likely to experience CHE than those households containing only one disabled person.

**Table 3:** Factors associated with catastrophic health expenditure

Variable	Catastrophic Health Expenditure		
	Adjusted OR		95 % CI
Individual level			
Sex			
Male	1.00		
Female	0.86	0.53	1.39
Age			
20–39	1.00		
40–49	0.52	0.19	1.46
50–59	0.94	0.37	2.38
≥ 60	1.51	0.55	4.17
Education level			
Above university	1.00		
Middle/high school	0.82	0.31	2.18
Below elementary school	0.97	0.36	2.66
Marital status			
Married	1.00		
Single	0.47	0.19	1.14
Divorced or separated	0.54	0.29	1.01
Severity of disability			
Low	1.00		
High	1.30	0.87	1.94
Self-rated health			
Good	1.00		
Bad	1.85	1.07	3.22
Chronic disease			
No	1.00		
Yes	1.95	1.44	2.75
Household level			
Medical-aid			
Yes	1.00		
No	2.09	1.20	3.65
Equalized income			
Q4 (highest income)	1.00		
Q3	1.35	0.72	2.56
Q2	1.16	0.57	2.36
Q1 (lowest income)	1.41	0.74	2.68
Disabled			
1	1.00		
≥ 2	1.70	1.01	2.86
Senior citizen			
0	1.00		
≥ 1	1.59	0.96	2.62

*OR* odds ratio, *CI* confidence interval

We examined the effect of enrollment on the Medical-Aid program on CHE according to chronic disease and disability severity status (Table 4). The poor not enrolled in the Medical-Aid program were 2.4 times more likely to experience CHE than the Medical-Aid enrollees, among households having chronic diseases. In addition, the poor not enrolled in Medical-Aid were 3.6 times more likely to experience CHE than Medical-Aid enrollees, among households living with persons with severe disabilities.

**Table 4:** Effect of enrollment in medical-aid on catastrophic health expenditure according to chronic disease and disability severity status

Variables	Medical-Aid	Adjusted OR^a^	95 % CI	
Chronic disease				
No	Yes	1.00		
	No	1.62	0.50	5.20
Yes	Yes	1.00		
	No	2.36	1.26	4.41
Severity of disability				
Low	Yes	1.00		
	No	1.03	0.46	2.29
High	Yes	1.00		
	No	3.59	1.73	7.45

*OR* odds ratio, *CI* confidence interval

^a^Adjusted for sex, age, education level, marital status, self-rated health, equalized income, number of disabled persons, and number of senior citizens

## Discussion

About 4.2 % of the poor not enrolled in the Medical-Aid experienced CHE, representing a higher burden of medical costs compared with the figure of 3.2 % among the general population in South Korea [22]. The non-Medical-Aid group had much higher medical costs for their treatment versus the Medical-Aid enrollees, because there is no government support for those who live in poverty not enrolled in the Medical-Aid program. We also found that low-income people not enrolled in Medical-Aid were 2.1 times more likely to experience CHE after controlling for other confounding variables. These results provide evidence of a healthcare “blind spot” with respect to the excessive burden of medical costs among households containing persons with disabilities.

If we consider only the scope of subjects for application, then nobody is excluded from receiving medical security benefits in Korea. However, those who do not actually receive medical social security benefits represent an exception: the majority of the poor not enrolled in the Medical-Aid program are excluded because of the eligibility of the support obligor. Although their income level is lower than the minimum necessary for living, they do not qualify for support because the income of the support obligor, who has a responsibility for their care, is above the threshold level. Thus, the government needs to expand medical assistance for households containing persons with disabilities. In 2014, the number of registered disabled is 2,494,460 and 15.7 % of them is Medical-Aid. Given yearly average total medical costs of Medical-Aid with disabilities is about $3,262, Korean government needs additional expenses for about 6.9 billion dollars if government covers all disabled persons in Korea [23]. Although the government has implemented medical support services, the selection criteria and range of benefits remain insufficient. Medical assistance services for persons with disabilities support only enrolled persons with disabilities according to the national Basic Livelihood Security System (poverty level ≤ 100 %) and the near-poor (100 ≤ poverty level ≤ 120 %). As mentioned above, many poor persons with disabilities below the 120 % poverty line are, in fact, excluded from medical support services. In addition, the proportion of the expenses of uninsured patients that hospitals in South Korea can cover, compared with their total medical expenses, is very low. For such patients, the high costs of treatment may be a barrier to their accessing needed health services. Thus, policymakers need to expand the provision of Medical-Aid to persons with disabilities by relaxing the strict criteria with respect to the support obligor.

Health-related factors affecting CHE in our study included self-rated health and chronic those, similar to the results of previous studies on persons with no disability [24, 25] and persons with a disability and chronic diseases [26–28]. If a household includes a person with chronic diseases, they would be likely to continually incur medical expenses that may be increased by expenses not covered by health insurance. In addition, in the present study households containing more than two disabled people were 1.7 times more likely to experience CHE than those households containing only one disabled person. This result is similar to those of previous studies; policymakers must consider the number of persons with disabilities in each household to improve policies [10, 29].

We also examined whether medical security was associated with CHE in households containing persons with disabilities according to chronic disease and disability severity status. The poor not enrolled in the Medical-Aid program were 2.4 times more likely to experience CHE than were the Medical-Aid enrollees, among households containing persons with chronic diseases. Persons suffering from chronic diseases have a greater need for medical services, such that their OOP spending, on chronic disease treatments, may be relatively higher than in healthy people. In addition, the poor not enrolled in Medical-Aid were 3.6 times more likely to experience CHE than Medical-Aid enrollees, among households containing persons with severe disabilities. Because persons with severe disabilities need more medical treatment and rehabilitation, the burden of healthcare services is correspondingly greater than for persons with mild disabilities.

This study had some limitations. First, we could not assess whether the subjects were Medical-Aid enrollees with high accurately, because our data did not index the different types of health insurance. Thus, persons with disabilities may have been enrolled in Medical-Aid by routes other than via NBLSS. However, most Medical-Aid cases are selected by NBLSS, and the proportion of Medical-Aid enrollees among our total population was similar to that of a previous study [17]. Second, it was difficult to determine the levels of CHE for persons without a disability, because we analyzed households containing persons with disabilities only. However, we did compare our results with those from previous studies on persons without a disability. Third, the actual prevalence of chronic diseases is likely higher than that reported in PSED, because some conditions may not have been diagnosed. Finally, we did not consider information for type of chronic disease due to limited data. Because some chronic diseases may likely influence the health services utilization than others, caution is needed in interpreting the results.

## Conclusions

Our study showed that the poor not enrolled in the Medical-Aid program were more likely to experience CHE than Medical-Aid enrollees, among households containing persons with disabilities. Given the additional expenses for treatment and rehabilitation associated with disability-related health problems, persons with disabilities are more likely to face barriers to needed medical services. Thus, policymakers need to relax the eligibility criteria for Medical-Aid for persons with disabilities.
